# Personalizing Nutrition Strategies: Bridging Research and Public Health

**DOI:** 10.3390/jpm14030305

**Published:** 2024-03-13

**Authors:** Vicente Javier Clemente-Suárez, Helia Carmen Peris-Ramos, Laura Redondo-Flórez, Ana Isabel Beltrán-Velasco, Alexandra Martín-Rodríguez, Susana David-Fernandez, Rodrigo Yáñez-Sepúlveda, José Francisco Tornero-Aguilera

**Affiliations:** 1 Faculty of Sports Sciences, Universidad Europea de Madrid, Tajo Street, s/n, 28670 Madrid, Spain; vctxente@yahoo.es (V.J.C.-S.); josefranciso.tornero@universidadeuropea.es (J.F.T.-A.); 2 Grupo de Investigación en Cultura, Educación y Sociedad, Universidad de la Costa, Barranquilla 080002, Colombia; 3 Faculty of Biomedical and Health Sciences, Clinical Odontology Department, Universidad Europea de Madrid, Tajo Street, s/n, 28670 Madrid, Spain; helia.peris@universidadeuropea.es (H.C.P.-R.); susana.david@universidadeuropea.es (S.D.-F.); 4 Department of Health Sciences, Faculty of Biomedical and Health Sciences, Universidad Europea de Madrid, Tajo Street, s/n, Villaviciosa de Odón, 28670 Madrid, Spain; lauraredondo_1@hotmail.com; 5 Education Department, Universidad Antonio de Nebrija, 28240 Madrid, Spain; abeltranv@nebrija.es; 6 Faculty of Education and Social Sciences, Universidad Andres Bello, Viña del Mar 2520000, Chile; rodrigo.yanez.s@unab.cl

**Keywords:** diet, public health, dietary patterns, dietary behaviors, microbiota, hormonal disruption, inflammation, cardiovascular health, metabolic health, mental health, oncology, dental health, visual health

## Abstract

In recent years, although life expectancy has increased significantly, non-communicable diseases (NCDs) continue to pose a significant threat to the health of the global population. Therefore, eating habits have been recognized as key modifiable factors that influence people’s health and well-being. For this reason, it is interesting to study dietary patterns, since the human diet is a complex mixture of macronutrients, micronutrients, and bioactive compounds, and can modulate multiple physiological processes, including immune function, the metabolism, and inflammation. To ensure that the data we acquired were current and relevant, we searched primary and secondary sources, including scientific journals, bibliographic indexes, and databases in the last 15 years with the most relevant articles. After this search, we observed that all the recent research on NCDs suggests that diet is a critical factor in shaping an individual’s health outcomes. Thus, cardiovascular, metabolic, mental, dental, and visual health depends largely on the intake, habits and patterns, and nutritional behaviors. A diet high in processed and refined foods, added sugars, and saturated fats can increase the risk of developing chronic diseases. On the other hand, a diet rich in whole, nutrient-dense foods, such as vegetables, fruits, nuts, legumes, and a high adherence to Mediterranean diet can improve health’s people.

## 1. Introduction

In recent years, although life expectancy has increased significantly, non-communicable diseases (NCDs) continue to pose a significant threat to the health of the global population. According to the World Health Organization (WHO), NCDs are responsible for 71% of all deaths globally and 85% of deaths in low- and middle-income countries [1]. The contrast in dietary patterns between high-income and low/middle-income countries significantly influences the prevalence of NCDs. In high-income countries, the availability of processed and unhealthy foods contributes to diseases such as obesity and type 2 diabetes. Meanwhile, in low- and middle-income countries, lack of access to nutritious foods can lead to malnutrition, nutrient deficiencies, and an increased risk of NCDs. Addressing these disparities requires interventions tailored to each context, focusing on promoting healthy dietary patterns in high-income countries and improving food security and nutrition education in low- and middle-income countries. Recognizing and addressing these differences is crucial for effectively combating the growing problem of NCDs worldwide [1]. In addition, it is estimated that NCDs represent 60% of global health spending [2]. In Europe, it is estimated that NCDs represent around 80% of total health spending. In the United States, the direct annual cost of NCDs is estimated at more than USD 1.7 trillion [3]. Thus, with people living longer, policies and programs that promote “active aging” and “healthy lifestyle” are becoming increasingly necessary.

In recent years, the global burden of NCDs has been increasing, with diet being a significant contributor. According to the World Health Organization (WHO), unhealthy diets are one of the leading risk factors for global mortality, accounting for approximately 11 million deaths per year. Indeed, unhealthy eating patterns have been linked to a range of chronic conditions, such as obesity, type 2 diabetes, cardiovascular diseases, and some types of cancer [4]. Conversely, a diet that is high in nutrients and low in unhealthy components, such as saturated and trans fats, sugar, and sodium, has been associated with reduced risk of chronic diseases and improved overall health.

For this reason, health organizations come together every five years to review scientific evidence on diet and health, which is then used to inform the Dietary Guidelines [5]. Regarding this, dietary patterns refer to the types and amounts of food, beverages, and nutrients in people’s diets and how often they consume [6]. Various methods are used to study dietary patterns, such as a priori methods that are based on scientific consensus or evidence-based approaches, and a posteriori methods that group individuals based on non-overlapping patterns. Other methods can include hybrid methods, clinical trials, and observational studies on food [7].

What is clear is that the authors agree that eating habits and behaviors are a factor with an influence on the co-factors of pathological comorbidity which lead to NCDs [8]. The human diet, consisting of macronutrients, micronutrients, and bioactive chemicals, can influence several physiological systems, such as immune function, the metabolism, and inflammation. In recent years, there has been growing recognition of the importance of the gut microbiota, a diverse community of microorganisms that reside in the gastrointestinal tract, in mediating the effects of dietary patterns on human health [9]. The gut microbiota is known to play a crucial role in the digestion and metabolism of dietary components, as well as in the regulation of host immune and metabolic functions. Therefore, understanding the interplay between diet and microbiota is essential for developing effective strategies to promote health and prevent disease [9]. In this context, recent research has focused on identifying specific dietary patterns that can optimize microbiota composition and function, as well as exploring the mechanisms underlying the health benefits of these patterns. As such, diet is a major modulator of hormonal activity, as it provides the necessary nutrients and bioactive compounds for hormone synthesis and the metabolism [10]. However, emerging evidence suggests that certain dietary components, such as endocrine-disrupting chemicals (EDCs), synthetic or natural compounds that can mimic or interfere with the action of hormones, potentially disrupting the delicate balance of hormonal signaling in the body, may lead to hormonal disruption and adverse health effects [11].

Furthermore, all the recent research in NCDs suggest that diet is a critical factor in shaping an individual’s health outcomes. Thus, cardiovascular, metabolic, mental, dental, and visual health highly depends on the intake, habits, and nutritional patterns and behaviors [4,9]. Authors suggest that one of the leading causes of morbidity and mortality worldwide, cardiovascular disease (CVD), can be attenuated with the implementation of the Mediterranean diet, by including fruits, vegetables, whole grains, nuts, and lean protein sources, improving several cardiovascular risk factors, including blood pressure, cholesterol levels, and insulin sensitivity [4]. Metabolic health, which includes conditions such as type 2 diabetes, obesity, and metabolic syndrome, is also influenced by diet [12]. A diet high in processed and refined foods, added sugars, and saturated fats can increase the risk of developing these conditions. On the other hand, a diet rich in whole, nutrient-dense foods, such as vegetables, fruits, nuts, and legumes, can improve metabolic health [13].

Mental health is another aspect of health that is influenced by diet. Several studies have demonstrated the link between a healthy diet and a reduced risk of depression, anxiety, and other mental health disorders [14]. A diet rich in omega-3 fatty acids, antioxidants, and B vitamins has been shown to have beneficial effects on mental health outcomes. In addition, oral microbiota, and therefore dental health, is also influenced by the diet, since added sugars and acidic foods and beverages increase the risk of tooth decay and gum disease [15].

The health effects of dietary patterns and the consumption of micro- and macronutrients are extensive. Regarding visual health, a diet rich in antioxidants, such as vitamins C and E, beta-carotene, and lutein, has been linked to a lower risk of age-related macular degeneration (AMD), cataracts, and other visual disorders. Similarly, a diet rich in omega-3 fatty acids found in fish and nuts has been associated with reduced risk of certain eye diseases [16]. On the other hand, unhealthy dietary patterns and behaviors have been linked to an increased risk of visual disorders and cancers. For instance, a diet high in saturated and trans fats, as well as refined carbohydrates, has been associated with an increased risk of AMD, cataracts, and other visual disorders [17].

Given the significant impact of dietary patterns and behaviors on public health, there is a growing interest in understanding the mechanisms by which these factors influence these health outcomes. Therefore, understanding the holistic impact of dietary patterns on various aspects of health, including cardiovascular health, metabolic health, mental health, dental health, and visual health, among others, is crucial for promoting optimal health outcomes. Thus, this narrative review aims to provide a holistic and complete vision with state-of-the-art information of the impact of dietary habits on health. This information is intended to provide practical applications to improve not only life expectancy, but also the quality of years of life, shedding light on a growing public health problem.

## 2. Materials and Methods

We conducted a comprehensive literature search using primary and secondary sources, including scholarly papers, bibliographic indexes, and databases including PubMed, Scopus, Embase, Science Direct, Sports Discuss, ResearchGate, and the Web of Science. We utilized MeSH-compliant keywords like diet AND public health AND dietary patterns AND dietary behaviors, in addition to health-related factors such as microbiota AND hormonal disruption AND inflammation AND cardiovascular health AND metabolic health AND mental health AND dental health AND visual health AND oncology to maintain the literature’s relevance.

After reviewing numerous articles related to the keywords, a decision was made to eliminate content based on specific criteria. The search focusing on diet and public health yielded a total of 249,305 results. We then utilized the remaining keywords, identifying metabolic health as the most significant one, resulting in approximately 45,000 outcomes. Afterward, we included food patterns and acquired 2240 manuscripts. The authors of the review meticulously examined the titles and abstracts of all retrieved publications to confirm the appropriateness of the research included in the analysis.

Exclusion criteria were applied to exclude studies that utilized outdated data, resulting in a final selection of 1897 high-quality articles. Subsequently, papers on irrelevant topics, unpublished doctoral theses, and studies not in English were eliminated. After identifying the relevant research, the review writers individually collected data from the selected publications, resulting in a total of 420 high-quality items to be included in our review. This comprehensive method guarantees the reliability and credibility of the data in this review.

The review authors engaged in collaborative discussions to merge their findings and produce the current narrative review. The reviewers worked together to merge their expertise and viewpoints, leading to a comprehensive analysis of the literature and a cohesive, informative narrative that aligned with the study’s objectives.

## 3. Diet and Microbiota

The gut microbiota is an ecosystem of microorganisms that colonize the digestive system of mammals, ranging from the stomach to the colon. It is a complex and dynamic network of microbes, consisting of about 500 to 1000 different species, and the total number of cells in this ecosystem is estimated to be about 10^14^ cells, which is ten times more than the total number of cells in the human body. This super-organism comprises both eukaryotic and prokaryotic cells that work together to maintain the overall health of the host organism. Its composition is unique to each individual and varies over time, influenced by factors such as genetics, diet, age, medication, and environmental exposure [18].

Its development begins at birth, with the establishment of the initial microbial community being determined by the mode of delivery. Infants born vaginally acquire a diverse microbiota, dominated by *Lactobacillus*, *Prevotella*, or *Sneathia* species, while those born by Cesarean section have less diverse microbiota, with a predominance *of Staphylococcus*, *Corynebacterium*, and *Propionibacterium* [19]. During childhood, the microbiota continues to evolve and diversify, eventually stabilizing into the adult microbiota, which is characterized by the presence of seven to nine phyla from the Bacteria domain. The adult microbiota is dominated by two phyla: Firmicutes and Bacteroidetes, which make up about 90% of the total phylotypes. The remaining minority groups belong to the *Proteobacteria*, *Actinobacteria*, *Fusobacteria*, and *Verrucomicrobia phyla* [20].

### 3.1. Unveiling the Crucial Link: Nutrition, Gut Microbiota, and Health

Recent research has shown that the gut microbiota plays a crucial role in maintaining overall health and preventing various diseases, such as obesity, diabetes, inflammatory bowel disease, and even mental health disorders [21]. And yet, there are two main modifiers of the microbiota, which are physical exercise and nutrition, the latter being the one that has the greatest impact on it. In this line, nutritional patterns and behaviors play a crucial role in determining the diversity and composition of our gut microbiota, as evidenced by various studies [22,23,24]. Despite the formation and function of gut microbiota being established early in life, it can be modified by our dietary choices and the specific types of foods we consume, as well as the specific bacterial species that colonize our gut.

In the earliest stages, despite bacterial colonization being due to the type and mode of birth, feeding the baby can lead to a subsequent change in its microbacterial composition. In this line, research has shown that formula-fed infants have a lower abundance of beneficial bacteria, such as *Bifidobacteriaceae*, and a higher abundance of harmful bacteria, such as *Enterobacteriaceae*, compared to those who are breastfed during the neonatal period [25]. As a result, breastfeeding may have a protective effect against becoming overweight or obese, while formula feeding appears to stimulate microbiome changes that are associated with being overweight or obese [26]. Even a brief exposure to formula milk during a hospital stay can lead to subtle differences in the gut microbiota composition [27].

Later, certain micro- and macronutrients are the ones that will have the greatest impact on the modulation of the microbiota. The intake of dietary fiber, a complex carbohydrate found in various plant-based food sources, is resistant to digestion and absorption in the small intestine [28]. Studies have shown that altering the dietary intake of fiber can significantly impact the composition of gut microbiota in individuals, with high-fat/low-fiber diets resulting in different microbiota compared to low-fat/high-fiber diets [29]. Additionally, the type of enterotype present in an individual’s gut microbiota is associated with specific dietary patterns, such as Bacteroides being linked to protein and animal fat consumption, and *Prevotella* being linked to carbohydrate and monosaccharide consumption [30].

### 3.2. The Quality of Diet: Straight to Modulating the Composition of Microbiota

Regarding the amount of nutrients, a randomized controlled study investigated the effects of increasing dietary fiber while maintaining energy and nutrient intake in two groups [31]. The group on a high-fiber diet had significantly lower levels of HbA1c and body weight compared to the control group. This suggests that dietary fiber promotes the growth of acetate- and butyrate-specific bacteria, leading to the increased production of short-chain fatty acids (SCFAs). This increase in SCFA production can stimulate the production of GLP1, a hormone that promotes insulin secretion, improves glucose homeostasis, reduces inflammation, increases satiety, and alleviates type 2 diabetes mellitus [32]. Thus, including enough dietary fiber in our daily diet can play a vital role in promoting the growth of beneficial gut bacteria, maintaining overall health, and preventing chronic diseases such as type 2 diabetes mellitus [32].

The type of protein consumed has a significant impact on the composition of gut microbiota. For instance, protein from red meat and dairy products can increase the abundance of biliary anaerobes and Bacteroides in the gut [33]. However, the fermentation of animal protein can lead to a reduction in Bacteroide abundance and short-chain fatty acid (SCFA) production, which triggers an intestinal inflammatory cascade and worsens chronic diseases [34]. Nevertheless, the consumption of specific animal or plant proteins has beneficial effects on gut microbiota by increasing the abundance of symbiotic Bacteroides and short-chain fatty acids [35].

A study involving patients with type 2 diabetes mellitus and non-alcoholic fatty liver disease (NAFLD) showed that consuming an isocaloric diet with a similar macronutrient content rich in either animal or plant protein for six weeks resulted in a significant reduction in insulin resistance (IR) and markers of necrotizing liver inflammation. In another study, obese patients with NAFLD were given isocaloric low-carbohydrate diets with increased protein content [36]. The liver transcriptomic analysis showed an increase in *Streptococcus*, which produces folic acid, and an upregulation of the folate-mediated single-carbon metabolism and fatty acid oxidation pathways. Therefore, the type of protein consumed plays a crucial role in determining the composition of the gut microbiota and its impact on overall health. While certain proteins like red meat and dairy products can lead to negative effects on gut health, the consumption of specific animal or plant proteins can have beneficial effects on gut microbiota and prevent chronic diseases.

The amount and quality of dietary fat have a significant influence on the composition of the gut microbiota [37]. Consuming a high-saturated fatty acid diet can lead to an increase in sulfate-reducing bacteria, resulting in defects in the mucous layer and heightened intestinal inflammation. Following a Western diet can lead to a significant decrease in gut microbiota diversity, impacting the essential microbial oscillators and disrupting the host’s circadian rhythm and metabolism, which can promote obesity [38]. Furthermore, the Western high-fat diet is rich in choline, which can be transformed by the gut microbiota into trimethylamine-N-oxide (TMAO), promoting the development of cardiovascular diseases [39]. It is essential to consider the effects of dietary fat on the gut microbiota, as it can have long-term impacts on overall health. A high-saturated fatty acid diet and the Western high-fat diet can increase the risk of intestinal inflammation and promote obesity, respectively. Moreover, the Western high-fat diet’s effect on TMAO levels can lead to the development of cardiovascular diseases [40]. Therefore, it is vital to maintain a balanced and healthy diet to ensure a healthy and diverse gut microbiome.

Not only micro- and macronutrients have an effect and impact on the modulation of the microbiota, but also elements such as probiotics and prebiotics [41]. Probiotics, which are live microorganisms found in many foods, can have beneficial effects on the host’s intestinal balance. Similarly, prebiotics, which are non-digestible food ingredients such as fructooligosaccharides and lactulose, can promote the growth of beneficial bacteria in the gut when used in combination with probiotics, known as synbiotics [42]. A recent review suggests that the use of two commercial probiotics can improve severe obesity in adolescents by reducing fasting blood glucose levels and the gut microbial Firmicutes/Bacteroidetes ratio. In prediabetic individuals, other commercial prebiotic brands have shown to improve metabolic disorders by altering the composition of the gut microbiota, resulting in a decrease in HbA1c levels. However, studies have shown that synbiotics may not always have a synergistic effect, as certain probiotic strains and prebiotics may compete with the permanent members of the microbiota [43].

### 3.3. Physical Activity: The Other Factor of Modulation

If we add physical exercise to the modulating factors of nutrition, we will find a perfect microbial balance according to the authors. The authors showed that endurance exercise in mice increased the abundance of beneficial bacteria, including Bifidobacterium and Akkermansia, while reducing the abundance of harmful bacteria such as Clostridium [44]. This shift in the microbial composition was associated with improved intestinal barrier function and reduced inflammation. In humans, professional rugby players presented a more diverse microbiome than sedentary individuals. The rugby players also had a higher abundance of butyrate-producing bacteria, which are associated with improved gut health and metabolism [45]. Furthermore, when physical activity is combined with prebiotic supplementation, it has been shown to reduce BMI, decrease liver enzymes and plasma cholesterol, and improve glucose tolerance in obese subjects and high-fat-diet-fed mice. Inulin, a type of prebiotic, has been found to regulate the growth of *Bifidobacterium*, *Dialister*, and *Catenibacterium* genera in obese individuals when physical activity is combined [46]. This suggests that physical activity may enhance the beneficial effects of prebiotics on the gut microbiota and may open a door for improved modulation benefits when exercise is combined with appropriate nutritional patterns.

Yet, the exact mechanisms through which exercise modulates the gut microbiome are not yet fully understood. However, researchers have proposed several hypotheses. One hypothesis is that exercise-induced changes in metabolism and hormone levels can determine the gut environment, making it more conducive to beneficial microbes. For example, exercise has been shown to increase the production of short-chain fatty acids (SCFAs), which are produced by gut microbes and have anti-inflammatory properties. Another hypothesis is that exercise-induced changes in gut motility and transit time can affect microbial diversity and [47].

Thus, we can add that the “second brain” dubbed by the authors, highly involved in the development of NCDs, is modulated by nutrition, a highly modifiable factor, and modification is enhanced when exercise is added, another modifiable pattern [48].

## 4. Diet and Hormonal Disruption

**Figure 1:**
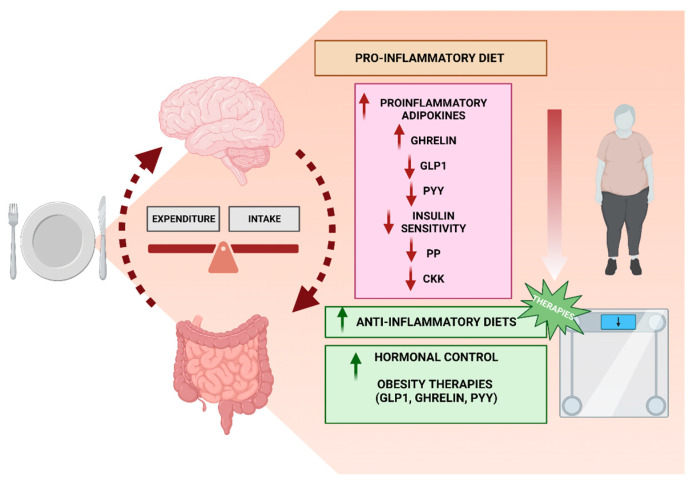
Influence of diet on hormonal functioning carried out by the brain–gut axis and the different strategies to achieve hormonal changes.

Diet is significant in terms of hormones because the energy and nutrients obtained from food serve as the basic materials for hormone production and body fuel. Hormonal fluctuations affect all of us at every stage of life, with varying effects between individuals [49,50]. Despite the widespread agreement that eating a healthy, well-balanced diet is crucial to our well-being, there is no universal agreement on how to achieve this. Hormonal imbalance and disease can be brought on by dietary variables such as food allergies, excess weight, a lack of exercise [51], a sedentary lifestyle, irregular sleeping and eating schedules, and digestive problems [50]. New evidence suggests that dietary components in circulation do more than only supply energy and micronutrients, but they may also activate receptors and signaling pathways. Thus, food may be thought of as a mixture of “hormones”. Throughout this process, it has been concluded that the brain–gut axis is a feedback loop that regulates our eating habits and other behavioral aspects of the nervous system (Figure 1). As a result, the health of this axis determines how our bodies process food and how our hormones react to it [52].

### 4.1. Deciphering the Interplay: Nutrition, Adipokines, and Hormone Sensitivity

Depending on dietary patterns or particular nutrients, nutrition may affect tissue or cellular receptivity to hormone signals in a variety of ways. These types of actions may be implemented directly or indirectly. Adipose tissue is an endocrine organ that dynamically secretes multiple hormones, the adipokines, that regulate these essential physiological processes [53,54]. Leptin, adiponectin, and resistin perform some of these vital physiological functions. In this regard, expanding adipose tissue undergoes a change in the adipokine secretion profile characterized by an increase in pro-inflammatory adipokines and a decrease in anti-inflammatory adipokines, resulting in an adverse circulating adipokine profile and a chronic, low-grade systemic inflammatory state [53]. Plasmatic levels of these adipokines, which are primarily derived from fat depots, are precisely regulated under various metabolic conditions, including obesity, fasting, diabetes, etc. [55,56]. For instance, different foods may either raise or reduce leptin sensitivity [57]. Saturated fatty acids, on the other hand, have been shown to induce leptin resistance by interrupting leptin signaling following chronic overstimulation of the leptin receptor [58,59], whereas anti-inflammatory diets like the Mediterranean diet and others rich in plant-based foods and healthy fats have been shown to decrease leptin levels and improve leptin sensitivity [60]. Thus, obesity may result from a general decrease in tissue sensitivity to leptin [58,61]. However, adiponectin raises the body’s rate of fat breakdown, speeds up the metabolism, and improves muscle’s capacity to absorb carbohydrates for energy [62]. Mozillo et al. reported that diet and exercise have been shown to significantly increase adiponectin levels [63]. It is known that the combination of a Mediterranean diet with caloric restriction and individualized counseling for aerobic exercise resulted in a 15% weight loss and a 48% increase in adiponectin levels [64]. However, adipokines-related pro-inflammatory processes may still have significant effects on the organism. Evidence reveals that leptin is a potential activator of reactive oxygen species (ROS) production in human mammary epithelial cells, where ROS production was apparently linked to NOX5 activation [65]. Hence, excessive nutrient intake, such as a high fat diet, may increase the production of ROS [66]. In turn, chronically elevated ROS levels may result in impaired insulin sensitivity [67,68] as well as impact on the modulation of thyroid hormones [69]. Thus, dietary patterns may influence leptin levels and sensitivity and any ensuing decreases in leptin signaling may influence thyroid hormone secretion, although the precise regulatory relationship is still unknown [70]. However, it has been widely believed that the administration of exogenous leptin, thereby increasing the hormone’s circulating level, should inhibit food intake and function as an obesity treatment strategy [71,72]. Evidence showed that the sensitivity of cells to hormonal signals may be modified by a patient’s physiological state, including systemic inflammation, visceral fat content, lifecycle stage, and glucose intolerance [73]. Although the involved inflammatory processes will be described in greater detail in subsequent sections of this review, it is well stablished that nutrition may indirectly influence the sensitivity of hormone signaling in a manner dependent on these states.

### 4.2. Appetite Regulation and Hormonal Dynamics

Evidence has recently reported how important it is for the brain and the body’s periphery to communicate in order to maintain proper appetite regulation. This calls for a nuanced interplay between environmental cues, hormones that both suppress and stimulate hunger, and the right kind of neuronal communication in the brain [74,75]. The mechanisms underlying nutrient-induced suppression of caloric intake are distinct for dietary protein and lipids [76]. High-fat, high-calorie diets impair the satiety-inducing effects of gastrointestinal hormones and, as a result, promote continued overconsumption [30]. These effects are mediated by alterations in signaling in both peripheral and central pathways, and dietary restriction may only partially reverse them. In addition to probiotics, meal-related factors (such as eating pace and frequency), circadian influences, gene polymorphisms, and additional factors influence energy intake and eating behavior [77,78]. Any deviations from this equilibrium can result in an imbalance between energy intake and expenditure, which frequently leads to excess and, in extreme cases, weight gain that may result in overweight or obesity [74].

To better understand the role that other key molecules like ghrelin, glucagon-like peptide-1 (GLP-1), peptide tyrosine-tyrosine (PYY), cholecystokinin (CCK), glucose, and insulin play in regulating appetite and satiety, neurochemical brain imaging studies have reached significant outcomes (Figure 1) [79]. As mentioned, by regulating dietary intake and energy expenditure, gut hormones and adipose tissue-secreted peptides are essential for the homeostatic regulation of body mass [50,79]. There is conflicting evidence regarding the relationship between obesity and levels of PYY and GLP-1, with some studies indicating that obese individuals have lower circulating levels of these hormones than normal-weight individuals, while others do not [80,81]. Coccurello et al. showed a high correlation between post-surgery weight loss and the hormones PYY and GLP-1 [80]. Likewise, obese adolescents and adults have lower levels of PYY in both fasting and fed states, indicating a complex deviation in the hormone’s homeostatic regulation [82]. This may be due to abnormalities in the synthesis, release, or excretion of PYY [83]. Nevertheless, multiple lines of evidence point to a connection between low circulating PYY concentrations and an increased risk of becoming overweight [84,85].

Moreover, pancreatic polypeptides (PPs) and ghrelin are two additional gastrointestinal hormones also altered in obese people [74]. Plasma PPs have been shown to be reduced under conditions associated with increased food intake; therefore, high-fat diets that possibly increase the need for higher intake given the type of nutrients ingested will cause subjects to have low PP values [86]. Also, findings suggested that ghrelin is lowered in women who have become accustomed to eating a low-glycemic-load diet [87]. In relation to the measures that can be taken in this respect, studies reported that PP concentrations were lower in obese children compared to their slender counterparts. In this regard, at the one-year follow-up, the PP levels of children who had lost weight had significantly increased [80,88]. Thus, the evidence points out that treatments such as the bariatric treatment sleeve gastrectomy reduces ghrelin levels, which in turn causes weight loss. However, measuring the active form of ghrelin in the plasma during fasting has significant caveats [89]. In contrast, Klementova et al. reported outcomes in adults that, after eating a single plant-based meal, the markers of the postprandial metabolism (such as GLP-1, amylin, and PYY) differed from those seen after eating a processed meat meal with an identical energy and macronutrient composition [90]. In this regard, postprandial plasma glucose homoeostasis, as well as GLP-1 and oxyntomodulin release, are all better with a Mediterranean diet meal than with a high-fiber vegetarian diet in type 2 diabetes overweight/obese participants [91]. Interestingly, the weight loss observed in animal models following repeated administration of PYY has sparked interest in developing these peptides as antiobesity treatments for humans [92]. Also, increased production of satiety hormones such as GLP-1, the decreased secretion of ghrelin, and increased thermogenesis are all possible processes by which high-protein diets contribute to weight loss (Figure 1) [93]. Nevertheless, to effectively combat obesity, it may be crucial to pinpoint the precise relationship between appetite and weight.

It has been shown that, during intense situations, the desire for sweet and fatty foods increases, likely due to their high reward value. The consumption of food increases cortisol levels [94]. Numerous studies have investigated the effect of the food macronutrient type on cortisol levels, but the results are unclear [95]. Studies have linked a Western-style diet, which is high in refined carbs and saturated fats and low in fiber, to an increase in cortisol release [96]. In contrast, a very-low-calorie ketogenic diet has a positive effect on the sympathetic nervous system (SNS) and hypothalamus–pituitary–adrenal (HPA) axis in the short term, playing an essential role in reducing stress and regulating cortisol levels in saliva for these reasons [97]. When all of these factors are considered, it has been demonstrated that stress has negative effects on the immune system. Changes in lymphocyte populations, the proportion of suppressor T cells, decreased lymphocyte proliferation, decreased numbers and activity of natural killer (NK) cells, altered NK cells, altered antibody response, and the reactivation of latent viral infections are examples of these alterations [98]. However, the equilibrium between the quantity and type of fat in the diet and the utilization of fat as a fuel during exercise may influence immune function [51]. Ascorbic acid’s effect on cortisol levels has been extensively studied [95]. In vitro studies have shown that vitamin C has no effect on cortisol synthesis induced by the adrenocorticotropic hormone ACTH [99]. Brody et al. demonstrated that 3000 mg of vitamin C per day leads to a speedier recovery of cortisol after an acute psychological stress response, but does not reduce the overall concentration of this hormone [100]. Numerous studies indicate that supplementation with high concentrations of ascorbic acid during the period preceding the start of endurance sports prevented the increase in cortisol levels. In addition, it reduced muscle pain and enhanced the efficacy of regenerative processes [101]. High concentrations of vitamins B1, B2, and niacin, which are involved in the metabolism and production of cortisol, produced a similar effect [102].

### 4.3. Impact on Pancreatic Function and Insulin Sensitivity

It has also been hypothesized that dietary composition can positively impact on pancreatic beta cell reactivity and, by extension, insulin sensitivity [103]. In 2021, for instance, researchers split 32 morbidly obese patients who were already on a bariatric surgery waiting list into two groups: one ate a low-calorie diet consisting of 30% carbohydrates, 30% protein, and 40% lipids, while the other followed a Mediterranean diet consisting of 55% carbohydrates, 15% protein, and 30% lipids. Both diets were found to be equally successful in reducing insulin resistance and increasing beta cell glucose sensitivity after four weeks of treatment, in addition to two sessions of behavioral dietary counseling, despite neither diet’s effect on fasting plasma glucose [104,105]. Thus, current guidelines recognize and encourage the use of low-carbohydrate diets for diabetes patients who are obese [106]. In this regard, Kiens et al. reported that, when consuming a diet with an energy composition typical of Western countries, transitioning from high- to low-glycemic-index carbohydrate sources decreased the insulin action on whole-body glucose disposal at a high but not a physiologic plasma insulin concentration [107]. Papakonstantinou et al. pointed out that chrononutrition is a vital component of the metabolic process, pancreatic function, and hormone secretion [108]. Postprandial glycemia and insulin sensitivity appear to be largely influenced by consuming the majority of calories and carbohydrates at lunch and early afternoon, avoiding supper in the late evening, and maintaining a consistent number of daily meals and relative eating occasions. The sequence of meals and nutrients also play an important role, as low-density foods, such as vegetables, salads, and soups, ingested first, followed by protein, and then by starchy foods, reduces the glycemic and insulin response [109].

### 4.4. Sex Hormones and Metabolic Health

Regarding sex hormones, the growing obesity epidemic in Western civilization is mostly attributable to the increased consumption of simple sugars and high-fat food brought about by the Western-style diet and physical inactivity [110]; consequently, the metabolic diseases caused by overfeeding and age-related hypogonadism have become increasingly common as the average human life duration has been extended well past the reproductive years. An imbalance in sex hormones can lead to infertility, obesity, glucose intolerance, dyslipidemia, and increased hunger, all of which are serious health problems [111]. The metabolic and reproductive consequences of sex hormone-related diseases, such as low testosterone in males and natural menopause and hyperandrogenemia in women, may be improved with dietary restriction and weight control according to clinical and translational investigations [112,113]. A study carried out by Bennet et al. has shown that, by influencing the concentration of female reproductive hormones, dietary fat may potentially influence the development of breast cancer [114]. This research found that changes to the vegetarian or fish diet had little effect on total hormone concentrations in the diet; however, the vegetarian group experienced a significant decrease in estradiol levels. There was a positive correlation between prolactin and fat intake, a negative correlation between sex hormone-binding globulin (SHBG) and fat intake (especially cholesterol), and a positive correlation between prolactin and complex carbohydrate intake [114]. However, other studies suggest that the relatively low BMI of vegetarians and vegans causes minor changes in SHBG and postmenopausal estradiol, but that the composition of vegetarian diets may not have additional effects on these hormones [115]. More recent research found that premenopausal and postmenopausal vegetarians have higher SHBG levels, which may be partially explained by their higher fiber intake. This may partially explain the reduced risk of developing type 2 diabetes [116]. Recent studies demonstrate that that intermittent fasting reduces androgen markers (testosterone and the free androgen index (FAI)) while increasing sex hormone-binding globulin (SHBG) levels in obese premenopausal women [117].

In conclusion, a Western diet increases the plasma levels of sex hormones and decreases the concentration of SHBG, increasing the bioavailability of these steroids. The identical diet leads to modest levels of mammalian lignans and isoflavone phytoestrogens. These diphenolic compounds appear to impact on hormone metabolism and production as well as cancer cell proliferation via multiple mechanisms, making them potential cancer-protective agents [118,119]. Therapeutic intervention may be successful in re-establishing equilibrium if the links between diet and cellular sensitivity to the hormone signal are identified [120]. For instance, identifying new mechanisms such as leptin regulation at the level of the entire body should be the focus of future research in order to design novel drugs that reverse leptin resistance. Understanding the pathogenesis of obesity-related disorders and the regulation of energy homeostasis by leptin should therefore provide new treatment options for obesity [61].

## 5. Diet and Inflammation

### 5.1. Metabolic Inflammation: Diet, Oxidative Stress, and Health Implications

**Figure 2:**
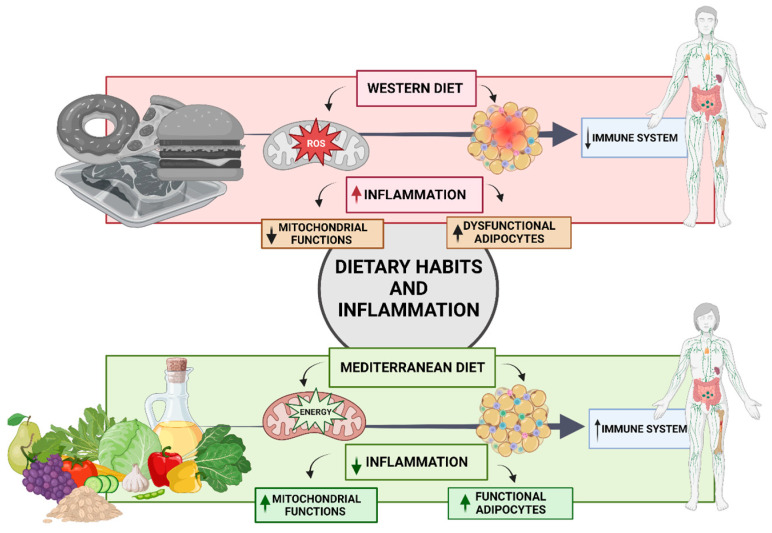
Differences between the Mediterranean and Western diets in their effects on inflammatory processes.

In Western societies, the consumption of calorically dense Western-style diets combined with chronic overnutrition and a sedentary lifestyle induces a state of chronic metabolic inflammation known as metaflammation [121]. It is the perfect combination that contributes to the worldwide increase in obesity, insulin resistance, and type 2 diabetes [73]. However, our understanding of how dietary intake causes the complex metabolic perturbations associated with obesity is limited. Multiple mechanisms, including oxidative stress, immune dysregulation, and intestinal microbiota dysbiosis, have been shown to contribute to the pro-inflammatory effects of a Western diet [120]. In contrast, interventions with an anti-inflammatory diet such as the Mediterranean diet (MD) led to a considerable decrease in body weight and visceral adipose tissue, as well as improvements in the cardiometabolic and inflammatory conditions of the participants. It has been demonstrated that the anti-inflammatory diet is effective for obesity management (Figure 2) [122].

The metabolic alterations associated with obesity are in part due to an accumulation of oxidative stress in the mitochondria, although the mechanisms and importance of this link remain unclear. White adipose tissue (WAT) fat cells’ endocrine and metabolic activity is impacted by mitochondrial oxidative stress and the formation of reactive oxygen species (ROS) [123]. The mechanisms of this metaflammation contributes to the development of numerous prevalent non-communicable diseases (NCDs), and these lifestyle-associated pathologies represent a growing public health issue with global epidemic proportions [124]. In this regard, evidence reveals that high-sensitivity C-reactive protein (HS-CRP), interleukin-6 (IL-6), and tumor necrosis factor alpha (TNF-a) have all been linked in human studies with assessments of habitual dietary intake as estimated by a food frequency questionnaire or 24 h recall [124]. Specifically, adherence to a Western dietary pattern, characterized by a high consumption of red and processed meats, refined cereals, and sugary beverages, was found to be associated with these well-established markers of inflammation (Figure 2) [125]. Likewise, excessive ROS production was linked to nuclear factor-B (NF-B)-mediated inflammation in obese individuals. Thus, recent research indicates that ROS play a role in the initiation, progression, and resolution of the inflammatory response [54,126]. NF-B controls the gene expression of multiple oxidant-responsive adipokines, including tumor necrosis factor-a (TNF-a) [66]. Moreover, AMP-activated protein kinase (AMPK), which is essential for energy homeostasis and the modulation of metabolic inflammation, can be downregulated by IκB-dependent TNF-a activation [66]. Conversely, Aleksandrova et al. provides evidence that plant-based dietary patterns are associated with reduced levels of oxidative stress and inflammation and may be an effective method for the prevention of chronic diseases [127]. Concretely, polyphenols, plant-derived chemicals, and polyunsaturated fatty acids are just some of the MD ingredients that have been shown in several experimental settings to boost mitochondrial metabolism, biogenesis, and antioxidant capability (Figure 2) [128]. Osorio-Conles et al. also reported that a short-term MD intervention was associated with the elevated expression of genes involved in adipogenesis, angiogenesis, and autophagy [129].

### 5.2. Harnessing Nutritional Anti-Inflammatory Power: Insights from the Mediterranean Diet and Plant-Based Diets

In contrast, adherence to a Mediterranean-style diet is strongly recommended due to its healthful dietary pattern, which includes antioxidant nutraceuticals such as polyphenols [84]. Most observational and interventional studies have found that the traditional Mediterranean diet, which typically has a high ratio of monounsaturated (MUFA) to saturated (SFA) fats and −3 to −6 polyunsaturated fatty acids (PUFAs), and supplies an abundance of fruits, vegetables, legumes, grains, and nuts, has anti-inflammatory effects [130,131]. Concretely, the positive effects of MD on blood pressure, insulin sensitivity, lipid profiles, lipoprotein particles, inflammation, oxidative stress, and carotid atherosclerosis were supported by significant improvements in traditional and emerging cardiovascular disease (CVD) risk factors [105,132]. Additionally, the vegetarian diet, the DASH diet, and the Paleolithic diet were associated with decreased levels of oxidative stress and pro-inflammatory biomarkers, and increased levels of antioxidant and anti-inflammatory biomarkers [133,134,135].

Given the preceding, inflammation is a multicellular organism’s protective response to injury in order to localize, eliminate, and remove toxic stimuli and recover (or replace) damaged tissues. Degenerative diseases like diabetes, cancer [136], cardiovascular [137], osteo-articular, and neurological diseases [138], autoimmune disorders [124], and aging are all caused by chronic inflammation, since it disrupts every physiological process in the body [60,66]. In particular, our understanding of how gene transcription factors contribute to inflammation is expanding. Many factors, including imbalances in hormones and gene expression, contribute to obesity, metabolic syndrome, and diabetes [139]. Furthermore, there is an inflammatory component to many diseases that may be affected by dietary changes [140]. Nutrient overload induces higher BMI and obesity, which is a state of chronic low-grade inflammation. The immune response can be modulated to a large extent by diet, so BMI may not be such a reliable benchmark. There is good evidence that low body weight makes people more susceptible to infection, a state known as immunosuppression. On the other hand, being overweight increases the risk of developing an inflammatory disease, which activates the immune system [141]. Antioxidant stress and immunological responses may benefit from vitamin and mineral supplementation. Inflammatory markers (CRP, TNF-a, and IL-6) have been shown to be correlated with vitamin and mineral intake in both cross-sectional and interventional investigations [142,143]. For optimal modulation of the immune system, as well as the regular permeability and activity of immunological functions, the Mediterranean diet remains the evergreen solution (Figure 2) [144].

Also associated with the Western diet is dysbiosis, or an imbalance of the intestinal microbiota, which can contribute to inflammation. Dysbiosis of the gut microbiota refers to an imbalance in the composition and function of the microbial communities inhabiting the human intestine [145]. Diets low in fiber and prebiotics are essential for the growth and diversity of beneficial gut flora [146]. When the diet is high in fat, sugar, and salt, this can instead encourage the development of pathogenic bacteria and fungi [147]. This can lead to intestinal inflammation, increased intestinal permeability, and the systemic release of bacterial endotoxins such as lipopolysaccharides (LPSs). These endotoxins can activate the immune system and stimulate the production of pro-inflammatory cytokines, including IL-1 and IL-18 [148]. In contrast, “Mediterranean” and vegetarian diets rich in fruits, vegetables, olive oil, and oily salmon are recognized for their anti-inflammatory properties and could prevent dysbiosis and subsequent inflammatory bowel disease [149].

The weight of the evidence suggests that diets high in ultra-processed foods are associated with an increased risk of developing a number of different chronic diseases and that diets that promote whole, minimally processed foods are essential for fostering gut health and general wellness. The results of this analysis show firstly how critical it is to take immediate steps towards regulating highly processed foods and, secondly, how modulating the immune system through nutrition may be an important technique for lowering the risk of non-communicable diseases, knowing that the evidence demonstrates that anti-inflammatory benefits may be experienced by persons who consume a diet high in plant foods and dairy products [150].

## 6. Diet Pattern and Cardiovascular Health

### 6.1. Cardiovascular Health Overview

Cardiovascular disease (CVD) is the leading cause of death in the world and affects both sexes equally [151]. In the last 20 years, the worldwide prevalence is estimated at almost 500 million people, doubling its value compared to previous years. The same is true for deaths from CVD, increasing from 12 million people in the 1990s to more than 18 million people in 2019, with more than 6 million deaths identified in people between 30 and 70 years of age due to these pathologies [152]. As for the disability associated with the presence of CVD, this has increased significantly, reaching 34 million people. Forecasts indicate that in 2030, almost 24 million people will die of CVD worldwide [153].

In this line, the increase in cardiovascular pathologies in recent years has been associated with the existence of risk factors such as the current lifestyle and the consumption of foods containing substances that are harmful to the body [154]. These factors facilitate the presence of adipose tissue in the abdomen, dyslipidemia, glucose intolerance, hypertension, etc., which increase the risk of the appearance of cardiovascular pathologies [155].

All these risk elements can be modulated to a large extent with a balanced diet and adequate nutritional patterns. However, it seems that the current trend indicates a change in the traditional diet in most countries, increasing the consumption of animal products, mainly red meat, and decreasing the intake of fiber present in cereals, fruits, and vegetables [156].

### 6.2. Toward Cardiovascular Wellness: Navigating Dietary Pathways for Health

However, more and more studies are showing the health benefits that appear to be determined by dietary and nutritional habits, and specifically on cardiovascular health [157]. Therefore, studies in this line are focusing on identifying the most valuable and the most harmful nutrients, and classifying the diets that are currently in use. In this sense, it is possible to identify some dietary patterns that have a direct impact on the appearance of these risk factors, and the compounds that determine the markers of metabolic and cardiovascular risk [158,159].

The Mediterranean diet has as essential characteristics the contribution of virgin olive oil as the main source of lipids in the diet, which is also complemented by the consumption of olives and dried fruits and seeds such as nuts, among others [160]. In addition, this diet has as its main base the consumption of whole-grain bread, pasta, and other unprocessed cereals, as well as vegetables and fruits that are rich in fiber, phytochemicals, and micronutrients. Fish is also present, as well as white meat and eggs as the main source of protein. Legumes are also included as a source of vegetable protein [161,162].

As mentioned above, this diet maintains a fat intake based on the consumption of olive oil, which contains a wide variety of phenolic acids, flavonoids, and secoiridoids, as well as active compounds such as coumaric acid, vanillic acid, tyrosol, luteolin, among others [163]. These compounds have an essential role in cardiovascular health due to their ability to lower blood pressure and increase blood flow in the coronary arteries [164,165]. Moreover, fiber plays an essential role in the prevention of pathologies such as diabetes, gastrointestinal diseases, and cardiovascular diseases. It is estimated that a daily intake of at least 30 g of fiber can lower risk markers such as blood glucose and cholesterol [166].

On the other hand, other diets that function as protectors in the appearance of cardiovascular risk factors have recently been studied. Among the most important, we find the DASH diet (Dietary Approaches to Stop Hypertension), initially designed with the purpose of reducing CVD; over the years, it has shown its efficacy in lowering blood pressure [167]. This diet has many factors in common with the Mediterranean diet, and a significant reduction in salt intake. It is rich in the consumption of vegetables and fruits, whole grains, fat-free or low-fat dairy products, nuts, and legumes [168]. In addition, the consumption of red meat and sugar-containing beverages is reduced. All this allows a high intake of fiber, potassium, and magnesium, which modulate hypertension and the glucose metabolism. On the other hand, the presence of polyphenols and antioxidants is favored, associated with adequate blood levels of glucose and insulin [169].

The vegetarian diet is also currently considered a good nutritional strategy in reducing the occurrence of CVD. It is mainly characterized by the elimination of the consumption of animal products and based on the intake of vegetables, fruits, legumes, cereals, and nuts [170]. In this sense, diets based on the intake of plants maintain a low energy intake and a high fiber content, which works as a preventive action against different pathologies [171].

The ketogenic diet, initially designed for the treatment of seizures in epilepsy, is now used for prevention and intervention in people with obesity and the prevention of CVD. In this case, it is a diet based on a high level of protein and fat, and a significant reduction in carbohydrates [172,173]. It is indicated to be applied with a precision that facilitates ketosis, i.e., the rapid production of ketones in the liver from fat accumulated in the body. This is a metabolic state that occurs when there is not enough glucose. For this to be possible, carbohydrate intake must be significantly reduced and protein and fat intake increased by the same amount [174].

Other types of diets, such as the Western diet or poorly regulated nutritional patterns, favor inflammation of the organism, and with it, the appearance of risk factors that trigger CVD [175]. In this regard, we know that the main risk factors are hypertension, obesity, and hypercholesterolemia [176]. To modulate them, low carbohydrate intake reduces obesity and improves atherogenic dyslipidemia, which considerably improves LDL cholesterol levels. The intake of polyunsaturated fatty acids (PUFAs), omega-3, omega-6, and omega-9, called essential fatty acids, have a positive response in inflammatory regulation and reduce cholesterol levels, triglycerides, and blood pressure, and decrease the risk of atherosclerosis [177,178].

In this line, dietary modification by substituting protein for carbohydrates has been shown to significantly reduce LDL cholesterol levels and increase HDL cholesterol. Similarly, fiber consumption also lowers LDL cholesterol, slowing glucose absorption and producing high hypocholesterolemic effects [179]. In addition, potassium intake should be considered, which is usually below the recommended intake, as well as calcium intake. Similarly, sodium intake should be reduced, not exceeding 6 g per day, distributed in all meals [156].

The impact of diet on cardiovascular disease and the appearance of risk factors that can lead to its presence and maintenance over time is evident. It is therefore essential to know the most appropriate nutritional patterns to ensure a good quality of life not only in patients with CVD, but also in the general population, to reduce the pathologies associated with dysfunctional eating habits for the organism.

## 7. Role of Dietary Pattern on Metabolic Health

Dietary habits may play an important role in overall health status, including metabolic health. Thus, different types of diets might produce diverse effects on the metabolic status, considering their quantity and quality regarding to macro- and micronutrients. In order to enhance the comprehension of metabolic pathologies, it should be considered that they include different metabolic conditions, such as insulin resistance, leading to type 2 diabetes mellitus, atherosclerosis and dyslipidemias, cardiovascular disease, and obesity, with them being interrelated and sharing common pathways [180,181]. Thus, previous authors suggested how these metabolic conditions may be affected by pro-inflammatory substances, triggering oxidative stress and inflammatory processes [182,183].

### 7.1. Metabolic Pathways

The conversion of fuel molecules’ chemical energy into usable energy is tightly controlled, and several factors impact on how various cells use glucose, fatty acids, and amino acids. Glycolysis, the metabolic pathway responsible for the conversion of glucose to pyruvate, takes place predominantly in the cytosol of cells in most organisms. The liberated free energy is utilized in the synthesis of adenosine triphosphate (ATP) and reduced nicotinamide adenine dinucleotide (NADH), both of which are high-energy molecules [184]. Fatty acid oxidation is a mitochondrial aerobic process that involves breaking down a fatty acid into acetyl-CoA units. Fatty acids progress through this process as CoA derivatives, employing NAD and FAD. Prior to oxidation, fatty acids undergo activation by using ATP in the presence of CoA-SH and acyl-CoA synthetase. The amino acid metabolism involves the biochemical processes responsible for the synthesis, degradation, and utilization of amino acids [185]. Amino acids are utilized by the body to synthesize proteins, enzymes, hormones, and several essential compounds [186]. Yu et al. found that following a healthy dietary pattern such as the Mediterranean diet or the Dietary Approaches to Stop Hypertension diet (DASH) may impact the glucose metabolism through circulating metabolites. This offers a new understanding of how diet influences the glucose metabolism and can help in developing strategies to prevent type 2 diabetes [187]. Additionally, a pattern characterized by higher levels of very-long-chain saturated fatty acids (VLCSFAs) and lower levels of alpha-linolenic acid (ALA) was linked to a healthier metabolic profile [188]. Research conducted by Bouchard-Mercier and colleagues indicates that the Western diet is linked to a distinct metabolic profile marked by elevated amounts of certain amino acids, such as branched-chain amino acids (BCAAs) and short-chain amino acids [189]; these conditions typically stem from inflammation and oxidative stress in the blood vessels, potentially leading to an increased risk of cardiovascular problems. Various signals influence cellular adaptation, including hormones that can rapidly alter enzyme activity and regulate gene expression to impact the cell’s metabolic profile. We need to comprehend metabolic pathways as interconnected processes that regulate and convert energy.

### 7.2. Metabolomic Profile and Diet Patterns

In this line, those diets that involve a large amount of refined sugar intake, such as the Western diet, may hurt metabolic health, since it has been pointed out that they may be the compounds of oxidative molecules that may compromise the metabolic status. In comparison to a high-glycemic-index (HGI) diet and a low-fat (LF) diet, the investigation of whether a low-glycemic-index (LGI) diet improved a collection of plasma metabolites linked to various metabolic illnesses was the objective of Hernández-Alonso et al. They reported that certain circulatory amino acid and lipid levels were modulated by an LGI diet. These results could explain the health advantages of low-glycemic-index diets in metabolic disorders like type 2 diabetes [190]. Galié et al. showed that adhering to the MD was linked to a certain plasma metabolomic profile that was also connected to improvements in the metabolism in adult metabolic syndrome patients [191]. Also, in a group that was typically healthy, plasma metabolite profiles linked to plant-based diets, particularly a healthy plant-based diet, were linked to a decreased incidence of type 2 diabetes [192]. It is important to note that exercise frequently and consistently modifies the average concentrations of metabolites associated with the energy metabolism and other branches of the metabolism in a variety of exercise styles and participants [193].

Regarding inflammation, different kinds of cytokines, including interleukin-6 (IL-6), C-reactive protein (CRP), tumor necrosis factor α (TNF-α), as well as adipokines, may be involved in inflammatory pathways producing obesity and insulin resistance, triggering systemic stress. Consequently, this might increase various signaling cascades, which may lead to atherogenesis as well as tissue fibrosis, compromising cardiovascular disease [194]. Additionally, a raised sugar intake may directly compromise type 2 diabetes, since diets which comprise of a low glycemic index have demonstrated their efficacy in controlling glycated hemoglobin levels and fasting blood glucose values compared to those diets which contain a high glycemic index in type 2 diabetes patients [195]. Thus, the recent literature proposed how type 2 diabetes mellitus risk may be related to a specific molecular pathway associated to Toll-like receptor 4 (TLR-4), generating insulin resistance [196]. Hence, it was described how TLR-4 may be activated through different substances, including heat shock proteins, fibronectin, fibrinogen, lipopolysaccharides, and different kinds of fatty acids, including free and saturated ones. Then, when this activation occurs, TLR-4 induces pro-inflammatory cytokine release, most of them associated to insulin resistance, promoting type 2 diabetes [197].

Regarding lipid intake, it has been largely described how those diets that include raised fat consumption also may be related to metabolic dysregulation and obesity [198,199]. Thus, previous authors proposed how a raised fat intake, more specifically saturated fat consumption, has been linked both to cardiovascular disease as well as to insulin resistance [200,201,202,203]. Furthermore, saturated fatty acids also may promote atherogenesis, triggering atherosclerosis events, which has been considered the most significant risk factor for cardiovascular disease and stroke [204,205]. As in the case of diabetes, inflammation and oxidative stress may constitute the most probable mechanism of action which could enhance metabolic dysregulation. In this line, the previous literature has highlighted the potential activity of saturated fatty acids in promoting postprandial inflammation [206]. The postprandial state happens when fatty acids and carbohydrates suffer an elevation in blood levels after eating, being a metabolic event that lasts between 6 and 12 h [207]. Thus, carbohydrates, especially refined sugar, as well as fatty acids promote inflammatory events, since both are considered as pro-inflammatory substances. Then, previous researchers proposed how fatty acids could have an analogous molecular pathway to lipopolysaccharides, leading to TLR-4 stimulation. Consequently, this promotes pro-inflammatory cytokine discharge, activating the immune response and enhancing metabolism disruption [208,209]. These results were consistent with the previous literature, wherein it was highlighted how the postprandial state may be associated to type 2 diabetes and atherosclerosis [210,211,212]. Additionally, regarding the fat quality, the previous literature noted how polyunsaturated fatty acids also may modulate metabolic diseases, since omega-3 polyunsaturated fatty acid intake was associated to a reduction in postprandial inflammation, while omega-6 polyunsaturated fatty acid consumption was related to inflammatory events [213,214,215,216,217,218]. In this line, the Mediterranean diet has been proposed as a healthy dietary pattern, since it implies a raised omega-3 polyunsaturated fatty acid intake, as well as the intake of many antioxidants present in fruits, vegetables, olive oil, and wine [219,220,221]. Furthermore, it has been largely proposed how the Mediterranean diet may constitute a protective tool against oxidative stress, since it is comprised of the intake of a large amount of antioxidant substances. Thus, it is composed of monounsaturated fatty acids and omega-3-polyunsaturated fatty acids, such as oleic acid and alpha-linolenic acid, respectively, which could be found in nuts and different kinds of fish, such as salmon, tuna, or sardines [222,223,224]. Moreover, several substances present in the Mediterranean diet have been proposed for their benefits in promoting health status. This is the case of polyphenols such as flavonoids present in fruits and vegetables, which have shown the capability to enhance health status and decrease inflammatory processes by reducing oxidative damage [225,226], a fact which may be positive for diabetes and cardiovascular disease development. For example, citrus flavonoids may decrease pro-inflammatory compounds such as TNF-a, cyclooxygenase-2, and nitric oxide synthase activity, which have been associated to inflammatory events [227]. Moreover, wine intake constitutes an important source of resveratrol, a polyphenol that has been related to inflammation, oxidative stress, and platelet aggregation inhibition [228,229,230], a fact which may decrease cardiovascular and metabolic risk. Regarding fiber intake, the previous literature suggested how diets that include a high fiber consumption, such as the Mediterranean diet, were related to a decreased cardiovascular and metabolic syndrome risk [231]. Thus, previous studies proposed how fiber may have a positive effect in both pathologies due to its ability to modify inflammatory and proliferation processes, events that occur at a gastrointestinal level. Then, the fiber ingested suffer a fermentation process developed by gut microbiota in the gastrointestinal tract instead of being digested. As a consequence, special kinds of fatty acids were generated as a result of this fermentative process, known as short-chain fatty acids, which have been highlighted as potential anti-inflammatory molecules [232,233,234].

### 7.3. Molecular Biomakers

In addition, finding dietary pattern-related molecular biomarkers (proteins and metabolites) may help clarify the biological processes behind the diet-related risk of chronic illness. Dietary biomarkers may also be linked to risk factors for chronic illness or to the presence of overt chronic illness itself. For instance, a distinct pattern of 52 proteins that enhanced pathways related to the cellular metabolism, hypoxia, inflammation, and atherosclerosis was linked to the DASH diet score. The DASH diet was effective in lowering blood pressure in clinical trials, and epidemiological studies have indicated that following the diet is linked to a lower risk of hypertension, CVD, and mortality [235]. According to earlier research, lipids make up a sizable fraction of the metabolites linked to dietary patterns. Sixty-three percent of the metabolites linked to the three dietary patterns were lipids [236]. We found that fats with ≥five carbon double bonds were generally linked to improved food quality. Furthermore, we found that blood triglycerides and the high-density lipoprotein metabolism were correlated with genetic variations (rs174548 and rs174550) linked to different highly unsaturated lipid species [237]. Future clinical interventions may be guided by proteins and metabolites associated with dietary patterns, which may have prognostic significance. To determine the genetic determinants of dietary correlates and investigate their potential relationship to the development of modifiable chronic disease, more research in both in vitro and in vivo settings is necessary.

### 7.4. Food and “Omics”

Functional foods have active components that can help prevent and manage chronic diseases like type 2 diabetic mellitus (T2DM) due to their physiological health advantages [238]. Regularly consuming functional foods may improve antioxidant, anti-inflammatory, insulin sensitivity, and anti-cholesterol activities, which are important for preventing and managing type 2 diabetes. The MD’s components, like fruits, vegetables, oily fish, olive oil, and tree nuts, are considered a model for functional foods because of its natural nutraceutical content, which includes polyphenols, terpenoids, flavonoids, alkaloids, sterols, pigments, and unsaturated fatty acids. Polyphenols found in the Mediterranean diet and polyphenol-rich plants like coffee, green tea, black tea, and yerba maté have demonstrated significant clinical advantages on metabolic and microvascular functions, reducing cholesterol and fasting glucose levels, and providing anti-inflammatory and antioxidant effects in high-risk and type 2 diabetes mellitus patients. Yet, the potential synergistic effects of combining exercise with functional food intake on metabolic and cardiovascular health advantages remain largely unexplored in individuals with T2DM and those who have undergone bariatric surgery. Functional dietary benefits can be identified using comprehensive biological profiling known as “omics”, which includes molecular, genetics, transcriptomics, proteomics, and metabolomics data. However, this approach is not well studied in multi-component interventions. To prevent and manage T2DM effectively, a tailored strategy should integrate biological and behavioral frameworks, incorporating nutrition education into lifestyle interventions for diabetes prevention. Functional meals can offer extra advantages in this approach [239]. In this regard, transcriptomics using DNA microarray technology is commonly used in food research due to its comprehensive expression data, well-established protocols, and high reliability and reproducibility compared to other omics methodologies. As a case in point, even minimal caloric restriction can induce expression of the cyp4a14 gene, suggesting that this gene may serve as a biomarker for the advantageous impacts of dietary factors on the energy metabolism. Also, proteome analyses involve the processes of protein separation, quantification, and identification [240]. As an efficient biomarker of the beneficial effects of food factors, prohibitin can be utilized. Also, the field of nutritional metabolomics exhibits potential in creating a reliable and impartial method for quantifying dietary intake [241,242]. Vázquez-Fresno, for instance, noted in the PREDIMED study that the metabolic fingerprints of the MD groups differed from those of the baseline and control low-fat diet groups [241].

## 8. Dietary Patterns on Mental Well-Being

### 8.1. Mental Health Overview

The increase in mental pathologies in recent decades has also increased interest in their study and etiology. Since the year 2000, there has been an increase in research into the causes of this significant increase in the prevalence and incidence of psychopathologies, and it has been found that diet has a high impact on the figures. It is currently estimated that at least 25% of the world’s population has or will have a mental disorder during their lifetime [242]. An important fact is that these pathologies significantly deteriorate the quality of life of people, and it is estimated that they die between 10 and 20 years earlier than the general population [243].

In this regard, the mental pathologies with the highest prevalence are mood disorders, specifically anxiety and depression. In 2019, around 1 billion people worldwide presented a mental disorder diagnosis [244]. As of COVID-19, these numbers have increased by 26–28% in just one year. Specifically, anxiety disorders affect more than 300 million people worldwide, with at least 58 million diagnoses in children and adolescents [245,246]. As for the prevalence of depressive disorders, according to the latest report by the WHO in 2022, there has been a 25% increase in diagnoses in the last two years, with approximately 280 million people worldwide suffering from this pathology [247].

### 8.2. Toward Mental Wellness: The Nutritional Paradigm Shift

This increase in diagnoses has led to a focus on the study of the factors that have an impact on their appearance and maintenance. Thus, it is known that in developed countries a large part of these pathologies is associated with environmental factors and certain lifestyle and nutritional habits [248]. In this sense, it is essential to maintain a balanced diet, as well as the way in which food is consumed. Today we know that the intestine–brain axis maintains a bidirectional communication through mechanisms such as the activation of the vagus nerve, the production of metabolites (short-chain fatty acids), the production of microbial antigens, and enteroendocrine communication through cell release [249,250].

These interacting processes modulate central physiological processes such as neurogenesis, neurotransmission, and neuroinflammation. It is now known that mental disorders, especially those classified as mood disorders, have a direct relationship with low-grade systemic inflammation, which is caused by alterations in the intestinal microbiota [251]. Precisely, recent studies in this line investigate the microorganisms that inhabit the intestine in order to determine which bacterial families are directly involved in the production of neuro-actives that are associated with memory, learning, and mood [252]. In this way, the aim is to know the most suitable microbial profile to maintain adequate mental health [253].

In this sense, we know today that there are certain nutrients that work because of their high antidepressant effect. Among the most important are iron, folic acid, omega-3 fatty acids, potassium, vitamins B6 and B12, zinc, and potassium [254,255]. In addition, the important role of polyphenols has been identified in reducing the symptoms associated with depression and in slowing down the cognitive deterioration present in this pathology [256]. Polyphenols have antioxidant, antilipidic, anti-inflammatory, and vasoprotective properties, among the most important. Foods rich in polyphenols are involved in the modulation of neurotrophin levels, neurotrophic factors essential in neuronal proliferation and found in olive oil and olives [257]. Cocoa is another nutritional source with nutraceutical and neuroprotective properties, although it should be consumed in its purest form [258,259]. This is important because cocoa is usually consumed with sugar, which is undoubtedly detrimental for anxiety and depression.

The same happens with the consumption of omega 3 fatty acids, since they are associated with the improvement of cognitive functioning, as well as for their capacity to fight inflammatory pathologies [260]. The group of B vitamins or thiamine is also essential in cognitive functioning, improving memory and the learning process [261]. Folic acid, on the other hand, is involved in the synthesis of amino acids, which makes it essential in the process of cognitive development [262]. Choline promotes a strong neuronal connection and also improves cognitive performance and memory [263]. Tryptophan is a metabolic precursor of melatonin, serotonin, and niacin, and people suffering from depression are known to be deficient in vitamin D3 and magnesium [264].

In this line, it is possible to state that the impact of diet on mental health is direct and there is now sufficient evidence to confirm that nutritional patterns can modulate mental health [265]. The cellular and neuronal networks that compose the intestinal microbiota are highly specialized and branch through the paravertebral sympathetic ganglia, sympathetic and parasympathetic outflow, and brain centers [266]. The enteric nervous system (ENS) is similar in structure to the brain neurons, and its functions are integrative, promoting transport through the blood circuit and mucosal lining. Substances such as melatonin, dopamine, GABA, vitamins, and 95% of the body’s serotonin, a mood-modulating neurotransmitter, are synthesized here [267].

Similarly, it is known that patients with mood disorders and other psychopathologies are more likely to have metabolic mutations, requiring higher levels of folic acid than the general population [268]. This warns about the need to improve the nutritional bases of these individuals, especially when they are being treated with pharmacology. On the other hand, these patients usually present a higher prevalence of comorbidity with other physiological pathologies, such as cardiovascular disease or diabetes, which considerably reduces life expectancy [269]. This is partly explained by the acquisition of unhealthy eating habits and also by the side effects of psychotropic drugs, which favor weight gain and, with it, the appearance of pathologies associated with obesity [270].

In this sense, it is essential to carry out measures that favor a correct diet to minimize the harmful effects of poor nutrition, which, in addition to facilitating the presence of organic pathologies, will explain the maintenance and aggravation of psychiatric and psychological symptomatology. For this reason, in recent years, research has been carried out on the psychobiotic properties of prebiotics and probiotics in order to include these products in the regular diet of people with psychopathologies [271]. These products regulate the intestinal microbiota and reduce the possibility of metabolic and inflammatory alterations [272]. Among the most prominent are the *Lactobacillus casei* strain Shirota, which reduces anxiety levels and improves overall mood, the formula *Lactobacillus helveticus* and *Bifidobacterium longum*, which reduces psychological anxiety, and B-GOS, which improves cognitive functioning and reduces cortisol levels [273,274].

There is an urgent need to find solutions that are easy to apply in people’s daily diet and that allow an improvement in the quality of life and health of people with psychological and/or psychiatric pathologies. Therefore, the approach to interventions should be multifactorial and consider the biochemical profile of patients, looking for nutrients that modulate brain activity and mood.

## 9. The Impact of Dietary Patterns on Cancer Development

The influence of diet pattern has been largely studied by several authors, since dietary habits may modulate cancer events and could be considered as a useful tool in tumoral disease prevention and management [275].

In this line, several researchers highlighted how cancer may be modulated through different molecular procedures, such as oxidative stress and inflammation [276]. Then, it has been proposed how tumoral events may be triggered by an increased production of reactive oxygen species (ROS), leading to raised oxidative stress negatively affecting different kinds of cells [277]. Consequently, different oncogene promotion may be increased, and a reduction in tumor suppressor levels may also occur, as has been described in the case of p53 levels, known for its capability to decrease cancer development. Furthermore, ROS activity also may be related to inflammatory processes, since it has been associated to raised cytokine levels [278]. According to these findings, the previous literature highlighted how a relationship may exist between cytokine production and tumor proliferation, wherein cancer progression could be affected [279,280].

### 9.1. Exploring the Link between Dietary Habits and Cancer: Insights into Macronutrient Quality

Regarding macronutrient quality, it has been mainly researched in order to elucidate which dietary habits may present a beneficial or detrimental impact in cancer management. For example, a study conducted in breast cancer patients showed how the presence of cancer events may be related to simple sugar consumption developed by those patients [281], suggesting the importance of high-glycemic-index carbohydrates in cancer progression. Furthermore, previous researchers investigated cancer incidence in Europe, suggesting how a positive relationship may exist between refined sugar consumption and colorectal cancer incidence [282]. These results may be explained by the Warburg effect, a process which has been extensively described by the recent and previous literature; Otto Warburg was the pioneer figure who first proposed this metabolic success [283]. Then, it has been suggested how tumoral cells may increase their growth and proliferation when they are surrounded by high glucose levels, constituting a nutrient-rich environment which may enhance the obtainment of energy through aerobic glycolysis [284]. This theory is also supported by the recent literature, wherein high glucose levels might be linked to a vascular endothelium growth enhancement, in addition to a reduction in anti-angiogenic elements [285], which could improve cancer development. Relating to cancer prognosis, several researchers associated a poorer efficacy in cancer treatment, also presented by patients with higher simple carbohydrate intake [286,287,288,289,290]. Thus, according to these findings, it could be considered that those dietary patterns which involve a raised refined sugar intake may be related to a higher risk of cancer development. In line with nutrient intake, the recent literature proposed a new tool in cancer management, since autophagy as well as intermittent and periodic fasting may have a positive effect on tumor incidence, as it has been described how periodic cycles of fasting mimicking diet may reduce different metabolic compounds, such as blood glucose, insulin-like growth factor 1 (IGF-1), and insulin. Then, these modifications may reduce inflammation, leading to an increase in immune system activity, as well potentially constituting a favorable tool in order to avoid DNA injury [290,291], thus preventing cancer promotion.

### 9.2. Lipid Quality and Cancer Progression

Regarding lipid consumption, the current literature highlighted how their quality also may have an important impact on cancer progression. Thus, the positive effect that omega-3 polyunsaturated fatty acids may represent in cancer disease has been suggested, since they have been pointed out, due to their anti-inflammatory effects, as useful elements in reducing oxidative stress and inflammatory events. Then, it was proposed how those patients that presented with a reduced omega-3 polyunsaturated fatty acid intake showed a 30% higher likelihood of dying of cancer [292,293]. Furthermore, a recent meta-analysis suggested how those patients who presented elevated docosahexanoic acid (DHA) and eicosapentaenoic acid (EPA) consumption, both considered as omega-3 polyunsaturated fatty acids, may be less likely to suffer from colorectal cancer [294]. Regarding the other types of fatty acids, the latest literature proposed how trans fatty acid intake could have a negative impact on cancer disease due to its pro-inflammatory effect inducing cytokine liberation, including tumor necrosis factor-α (TNF-α), interleukin-6 (IL-6), and monocyte chemoattractant protein [295,296]. These findings were also in accordance with the recent literature, which described how colorectal cancer may be linked to increased trans fatty acid consumption [297]. Then, it could be considered how those dietary habits which involve better lipid quality, avoiding saturated fatty acids and trans fatty acids, known by their potential inflammatory process’s activity, may be helpful in a reducing cancer progression.

### 9.3. Protective Role of Fiber Consumption in Cancer

Dietary habits which involve fiber consumption also have been related to a reduction in different cancer comorbidities, as in the case of breast cancer patients. Then, it was described how breast cancer longevity may be increased in those patients who presented with a raised fiber intake [298]; moreover, it was pointed out by previous researchers how those patients who showed higher fiber consumption could be less likely to develop breast cancer [299]. These findings suggest the protective effect that fiber may have in cancer events. As mentioned above, this could be explained due to fiber not being digested in the gastrointestinal tube, and, consequently, being fermented by gut microbiota, resulting in an increase in short-chain fatty acid production, which reduces inflammatory processes. Additionally, fiber may increase the fecal bolus, increasing intestinal transit, and which therefore may favor the fecal elimination of carcinogenic substances [300].

Finally, as mentioned above, dietary habits that include significant antioxidant substance intake may constitute a helpful instrument which may reduce oxidative stress as well as inflammatory response, reducing cancer enhancement [301]. Thus, it has been proposed how polyphenols, terpenoids, and flavonoids found in fruits and vegetables, all of them known by their antioxidant properties, could decrease oxidative stress, thus being useful in cancer prevention [302,303]. Additionally, it has been suggested how polyphenols present in nuts may modulate the apoptosis process, as well as having a positive impact on cancer, since they reduce cell growth and migration, decrease angiogenesis processes, and reduce metastasis progression [304,305,306,307,308]. Finally, resveratrol, a polyphenol present in wine, has been elucidated by its capacity to reduce inflammation and oxidative stress, thus being useful in reducing the incidence and development of cancer disease [309].

## 10. Dietary Patterns on Oral Health

The oral cavity harbors a dynamic and abundant microbiota [310] that establishes itself as a highly organized biofilm associated with an extracellular matrix in which different types of microorganisms participate [311]. These microbial communities contribute to health by adjusting immune responses [312]. Periodontal disease and dental caries are the most prevalent polymicrobial etiology diseases worldwide. They are diseases that are not caused by the introduction of external pathogens to the oral environment, but by the disruption of homeostasis and the consequent dysbiosis that leads to changes in supragingival and subgingival communities associated with health states [313]. Both periodontal disease and caries are mediated by synergistic interactions within microbial communities that are influenced by the characteristics of available binding surfaces, oxygen availability, host exposure, saliva-segregated products to supragingival communities, and gingival crevicular fluid to subgingival communities [314]. These diseases are driven by diet and specific host behavior in the case of dental caries and immune system interactions in the case of periodontal disease.

In this sense, dental caries are lesions in the dental enamel that can affect the underlying dentin and develop as a result of microbial growth imposed by sugar in the diet and carbohydrate metabolism, which leads to localized acidification and the disruption of homeostasis in the dental mineralization process [315]. On the other hand, periodontitis is a chronic and progressive disease characterized by the expansion of dysbiotic microflora in the gingival margin, with the formation of an inflammatory infiltrate that contributes to the destruction of the connective tissue attachment to the tooth, resorption of the alveolar bone, and, consequently, the loss of dental support. It is also correlated with certain systemic diseases such as rheumatoid arthritis or cardiovascular diseases [316,317].

Nutrition is a key factor for the prevention and treatment of oral diseases, as it affects both the host’s health and the composition and metabolism of the oral microbiota. Evidence indicates that macronutrients and micronutrients can modulate pro-inflammatory and anti-inflammatory cascades, thus influencing the host’s microflora [318]. Therefore, it is recognized that the alteration of the composition of microbial communities is multifactorial and dynamic, promoting homeostasis or dysbiosis that influences health status.

### 10.1. Role of Oral Microbiota in Health

The microbiome performs various functions that are beneficial to the host, mainly in the intestine, establishing a balance between the host and the microbial community. However, there is also evidence of key positive functions of the oral microbiota according to studies in humans and mice [319].

Two hypotheses are proposed. The first is that the main function of the resident microbiota is to act as a physical and biochemical barrier to prevent colonization or infection by exogenous organisms, restricting access to the host epithelium and sequestering nutrients, making the pH acidic and microbicidal. The second hypothesis is that the function of the host microbiota is to promote the maturation of the innate and adaptive immune system to achieve an adequate balance between anti-inflammatory and pro-inflammatory processes in the absence of infection and during it. Hence, the relationship with the participation of neutrophils in periodontal disease microbiota [320,321]. Thus, we observe that there is a relationship between intercellular interactions and biological balance.

The host’s immune system is modulated by genetic, environmental, and behavioral factors, where oral hygiene, fluoride use, and dietary habits are of great importance. Therefore, it is important to observe how dietary patterns and diet characteristics influence the composition of the oral microbiota.

### 10.2. Characteristics of the Diet

It is important to analyze the risk factors that affect individuals’ health through diet in order to understand and modify them to improve health status. The consumption of fermentable carbohydrates in frequency and quantity is one of the drivers that negatively impacts oral health by causing tooth decay [322] and several chronic diseases, such as cardiovascular diseases, obesity, and various types of cancer [323,324]. Therefore, it is important to give importance to dietary patterns in which added sugar in beverages and food dominates over the intake of fruits and vegetables, as there is a direct association with the occurrence of caries due to structural changes in the microflora [325]. This is because carbohydrate consumption favors the growth of saccharolytic microorganisms, creating a favorable environment where they can become predominant over species with slower growth and alternative nutritional requirements [326]. Additionally, we observe how adherence to healthy dietary patterns with anti-inflammatory components that include a reduction in processed carbohydrates and an increase in fiber, vitamin C, and omega-3 fatty acids leads to changes in the composition of the oral microbiota, improving the composition and microbial interaction [327]. Similarly, we observe beneficial effects on the modulation of immune responses to gingival and periodontal inflammation due to the reduction in oxidative stress through increased antioxidant intake from the diet [328]. We observe a significant association with lower likelihood of being affected by periodontal diseases with greater adherence to dietary patterns such as DASH (Dietary Approaches to Stop Hypertension) diets, which aim to control blood pressure, and Mediterranean diets, which predominantly feature plant-based foods, fish, and olive oil [329].

### 10.3. Dietary Patterns

The pattern of food intake can also be considered a risk factor for the occurrence of dental caries. It is important to note that, in addition to the consumption of carbohydrates included in the diet, the consumption of these in the form of snacks and food between meals also affects the occurrence of dental caries. There is an increase in carious lesions in groups that exhibit this behavior pattern [330] and do not perform proper oral hygiene after intake [331]. Additionally, the time taken to consume meals also influences the development of caries. Individuals who take longer to eat have less capacity to satiate themselves, which can lead to snacking on foods with high sugar or calorie content and increase the risk of caries [332]. It is important to control these types of dietary patterns both in childhood and adulthood, as diets that include fruits, vegetables, and vitamins have been shown to reduce the association with the prevalence of caries compared to diets that are rich in fermented carbohydrates [333,334,335]. It is also important to be aware of intrinsic sugars in the diet, as there are many highly cariogenic foods that cause repeated drops in pH and favor the demineralization of dental tissues [336]. Therefore, a multifactorial approach that considers the importance of brushing, sugar intake, and proper oral education is crucial [337,338,339]. Similarly, the dietary intake of sugary beverages can have adverse effects, as there is a mixed composition in such drinks. Sugary beverages have been identified as drivers of caries risk, showing a clear dose–response relationship [340]. On the other hand, periodontal disease is inversely related to the consumption of coffee and milk but promoted by the consumption of alcohol due directly to the dysbiosis caused by its intake and indirectly to the alteration of the immune system [339,340].

Another habit that can influence oral microbial niches is smoking. It affects the composition of oral bacteria and can prepare the oral environment for the colonization of pathogenic bacteria that increase saliva acidity, deplete oxygen, and exhibit resistance to both antibiotics and the destruction of immune cells by the host [341], thereby potentiating the periodontal inflammatory microbiome [342]. This dysbiosis affects both the oral and nasal microbiome, as well as the oropharyngeal, pulmonary, and intestinal microbiome [343], leading to an environment that can become carcinogenic if levels of microbial metabolites progress [344].

The importance of achieving healthy dietary patterns is because intestinal flora imbalance can play a secondary role in the pathophysiology of various diseases, since this community performs several regulatory functions within the host organism [345]. One of these interactions occurs when there is potential inflammation in the diet, and chronic inflammation is enhanced by the prevalence of *P. gingivalis*, a bacteria involved in the process of periodontitis [346], resulting in the alteration of the intestinal barrier and bacterial translocation into the systemic circulation, leading to a risk of heart failure due to atherosclerosis [347]. These diets are also related to hypercholesterolemia and type 2 diabetes mellitus. The gut–brain–microbiome axis that influences central nervous system homeostasis can also be affected. There is bidirectional communication that affects both the immune and neuronal systems [348]. The intestinal mucosa is more permeable during an inflammatory state or dysbiosis, exacerbating neuroinflammation and causing neuronal impairment. Several microbiome-derived neurotoxins produced by intestinal and oral bacteria participate in the misfolding of Aβ, an oligomer that appears in the prodromal phase of Alzheimer’s disease (AD) in the extracellular space [349]. Although the association between periodontal disease and AD is not clear [350], dysbiosis is a relevant risk for AD, and high-quality diets may be associated with better cognitive capacity [351,352,353,354].

## 11. The Importance of Dietary Patterns on Visual Health

The concept of visual health refers to a state of well-being that allows an individual to see and perform daily activities without limitations and with maximized functional capacity. Visual health can be considered both a process and a result, and is dependent on environmental factors, thus it can be affected by various conditions that cause visual disability or vision impairment [355]. Vision is produced through the coordinated function of the accessory tissues of the eyes and the brain. The cornea and lens of the eyes focus light onto the photoreceptors of the retina and subsequently transform the light stimuli into neural impulses that lead to the formation of a three-dimensional image in the brain. Clear vision requires the structural and physiological integrity of all components of the visual system (eyes, brain, and its pathways), and any anomaly results in visual dysfunction. Several factors contribute to the development of visual dysfunction, such as genetic mutations, the environment, lifestyle, age, and malnutrition [356,357,358,359]. Individuals with visual dysfunction comprise all ages, with a higher prevalence in children and the elderly. Moreover, ocular diseases are more common in women, ethnic minorities, and individuals living in rural areas [360,361], and have social, psychological, and cognitive implications that affect the quality of life of the individual, increasing the need for social attention for these individuals [362,363]. Diet, as a determinant factor of lifestyle, can have long-term effects on ocular health and play a crucial role in the prevention of visual dysfunction, which can cause permanent visual disability or blindness and is estimated to affect one billion people worldwide by 2050 [364]. Additionally, there is a limitation that patterns are not usually maintained throughout the individual’s life, and there is a great interaction between genes and nutrients within the same genetic pattern. Therefore, it is important to observe the predictors and consider supplementation with individual nutrients or unique whole foods to improve the visual health of the individual [365].

The risk factors for visual dysfunction are multifactorial, and there is a global trend of an increasing adult population with visual impairment and dysfunctions caused by many ocular disorders, such as refractive errors, dry eye disease, age-related macular degeneration (ARMD), diabetic retinopathy, glaucoma, and cataracts [366]. Thus, we observe that loss of vision is a biomarker for a decrease in health span, and that there are behavioral patterns, including dietary habits, that can prevent or improve an individual’s ocular state [17,367].

One of the most important risk factors for ocular disorders is the degeneration of ocular tissues, which is sometimes influenced by aging. Therefore, it is necessary to understand the pathophysiology of these dysfunctions to employ preventive measures based on diet. Age-related macular degeneration involves damage to the macula, the area of the retina required for higher visual acuity. It has two forms of presentation. Dry ARMD is the most common and results from damage to support cells and is associated with extracellular deposits containing protein and lipids. Wet ARMD is less common but more severe and is the result of angiogenesis with fluid release, which results in the degeneration of photoreceptors [17,368]. Hence the importance of high-quality nutrients in our diet, so that the support cells do not become a risk factor [369].

The onset and progression of age-related macular degeneration is associated with low levels of carotenoids, antioxidant vitamins, omega-3 fatty acids, and a reduced intake of fruits, vegetables, and fish [370,371]. Additionally, it has been demonstrated that a high consumption of vitamin A, vitamin C, zinc, copper, and carotenoids could reduce the progression of ARMD by approximately 25% [372]. In the case of glaucoma, it is the loss of peripheral vision due to damage to the optic nerve and an increase in intraocular pressure. Although the most important risk factor is age, and there is no well-established association of nutrients and foods with glaucoma, cohort studies have shown that regular consumption of leafy green vegetables and those rich in nitrates, and the intake of vitamin A and C, are associated with a reduced risk of developing glaucoma [373,374].

Cataract is another common ocular disorder in people over 50 years of age, and it is an opacification of the lens. Although it is also related to age, a reduced risk of cataracts has been associated with the consumption of vitamin A, C, and E, lutein, zeaxanthin, and β-carotene [375].

Lastly, diabetic retinopathy is a microvascular complication caused by diabetes mellitus and is also associated with older age, longer disease duration, and poorer control of hyperglycemia [376,377]. The retina is susceptible to damage due to high glucose concentrations, as it is a metabolically active tissue, resulting in limitations in glucose transporters’ function, causing cellular damage and apoptosis through advanced glycation end-product (AGE) accumulation [378]. Therefore, regulating glucose levels through diet can be a preventive measure for diabetic retinopathy [17], as carbohydrates have different effects depending on their digestion and absorption rate. A carbohydrate that is rapidly assimilated produces a faster and more substantial increase in blood glucose and insulin. This increase in insulin creates resistance, leading to far-reaching effects, such as increased intestinal permeability [379], oxidative stress, and greater protein damage caused by AGEs [379], which have been linked to several diseases, including retinopathy.

### 11.1. Diet and Eye Health

The importance of maintaining a balanced and healthy diet that includes foods that promote the health of the ocular structures is fundamental in regulating their proper functioning. Certain foods, specifically micronutrients, have physiological effects that promote the onset of certain diseases [17]. Nutrient deficiencies can lead to malnutrition effects on the visual development of newborns and manifestations of ocular diseases in adults [355]. Therefore, a healthy lifestyle indicated by healthy eating habits is important. Adherence to certain types of diets promotes a decrease in risk factors for the onset of ocular disorders [380,381].

The Mediterranean diet is an example of a healthy dietary pattern, as it provides certain micronutrients, such as minerals, vitamins, carotenoids, omega-3 fatty acids (through the consumption of fish, olive oil, and nuts), which ensure the proper functioning of the visual system [382]. This diet has many anti-inflammatory and antioxidant factors due to the high consumption of fruits and vegetables, which reduce blood sugar levels. This diet acts on dyslipidemia, inflammation, oxidative stress, and hyperglycemia through some of its food components [383,384], reducing the incidence of various diseases. The association between the Mediterranean diet and age-related macular degeneration is beneficial, since the risks of progression of this disease are delayed due to the consumption of omega-3 fatty acids, especially fish consumption [385,386]. Recently, it has been observed that intestinal dysbiosis increases susceptibility to various ocular diseases, such as dry eye, uveitis, macular degeneration, and glaucoma [387]. This suggests the existence of a gut–eye axis (or gut–retina axis) mediated by the intestinal microbiota, since alterations in the intestinal microbiota cause many intestinal and extraintestinal diseases, including non-alcoholic fatty liver disease [388], inflammatory and metabolic diseases, obesity, and cancer [389]. Increased intestinal permeability allows for the excretion of endotoxins that activate inflammation in various tissues, extending to cells of the retinal pigment epithelium. Therefore, we can indicate that the Mediterranean diet modifies the intestinal microbiota in various ways by increasing microbial diversity [385], and there is a compelling link between the intestinal microbiota and ocular health [390]. The dietary pattern offered by the DASH diet is related to the nutritional management of hypertension and is also associated with anti-inflammatory and antioxidant properties [391], which improve glycemic and cholesterol control [392]. On the other hand, the low-calorie diet is another alternative that offers improvement with respect to visual dysfunctions, since calorie restriction slows the deterioration of ocular functions and improves mitochondrial function, but it should be under medical supervision, since rapid glucose reduction is associated with the worsening of diabetic retinopathy [393].

### 11.2. Natural Molecules in Food/Supplements That Impact Visual Health

Foods provide a variety of micronutrients, vitamins, and biologically active compounds that may play a fundamental role in visual system function [394], making it interesting to study the different nutrients and their mechanisms of action associated with a lower incidence of visual dysfunction [360].

#### 11.2.1. Omega-3 Fatty Acids

Regular consumption of foods rich in omega-3 fatty acids is associated with a lower incidence of age-related macular degeneration [353] and diabetic retinopathy [363]. These diseases are attenuated by the antioxidant and anti-inflammatory activity of these fatty acids, which also decrease the risk of diabetes by improving insulin sensitivity [395]. Essential omega-3 fatty acids obtained through a healthy diet or appropriate supplements provide benefits to the visual system by modulating systemic inflammation through the production of anti-inflammatory metabolites [396,397]. This mechanism can also help protect the optic nerve and regulate intraocular pressure by delaying and/or preventing glaucoma [379]. Additionally, long-chain omega-3 fatty acids are useful for reducing ocular surface inflammation in dry eye disease by improving the lipid profile of tears. Furthermore, diets with a high content of polyunsaturated fatty acids improve the cellular response of the retina in subjects with age-related macular degeneration against oxidative, ischemic, and inflammatory damage [398].

#### 11.2.2. Antioxidants

Dark green leafy vegetables and fruits contain abundant antioxidant nutrients, such as vitamin C, provitamin A carotenoids, and vitamin E, associated with a decrease in age-related macular degeneration in a large number of prospective studies [399]. Blueberries also have a high antioxidant capacity due to their abundant polyphenolic components and have been shown to be bioactive and beneficial to health. They affect visual health because the retina is an important source of oxidative stress and can retain amounts of anthocyanins that aid in this function. Several studies have shown that higher intake of flavonoids is associated with lower risk of cataracts, reduced risk in age-related macular degeneration, and improvement in individuals with diabetic retinopathy [400], but epidemiological evidence is lacking on the benefits of blueberries or anthocyanins on human vision [401]. Taurine is also an amino acid with antioxidant properties found in high concentrations in the mammalian retina [402]. This compound exerts a protective effect on the retina and photoreceptors against light-induced oxidative stress. Therefore, taurine supplementation could be a useful strategy to prevent or attenuate retinal degeneration associated with various ocular pathologies [403,404].

#### 11.2.3. Carotenoids: Lutein/Zeaxanthin

Lutein is a type of carotenoid found in many plant-based foods and plays an important role in the development and protection of the brain and eye, which are organs that share an embryonic origin. Lutein can be obtained from fruits, vegetables, and breast milk, so it is recommended to consume it during pregnancy and lactation [405]. Lutein has anti-inflammatory effects and also helps filter blue light that can damage vision and prevent chronic diseases [406]. It is regularly consumed in the human diet through fruits and vegetables [407], and its high intake, either through diet or supplements, has benefits for health, especially for ocular health and cognitive function [408], and influences some aspects of cardiovascular health, among other diseases [409].

#### 11.2.4. B-Complex Vitamin

The B-complex vitamins are a group of eight water-soluble vitamins that have coenzyme functions in various cellular metabolic reactions. Among them, vitamins B1, B2, B3, B6, B8, and B12 have been studied for their possible association with glaucoma [410]. These vitamins are involved in energy production, DNA and RNA synthesis and repair, and the synthesis of various neurochemicals, which contribute to the maintenance of brain function. In addition, vitamins B6, B12, and folic acid may have a protective effect against macular degeneration by reducing the plasma levels of homocysteine, an amino acid that can cause alterations in blood vessels when it is present in elevated levels [411]. Deficiency of B vitamins can negatively affect vision by compromising the optic nerve, which can cause a decrease in visual acuity [412,413].

#### 11.2.5. Calcium

Calcium is an essential micronutrient that participates in numerous vital physiological functions, and although most studies on the role of calcium have focused on bone health, attention has recently been paid to the effects of dietary calcium and calcium supplements on other health outcomes [414]. It has been suggested that dietary calcium is related to blood pressure by regulating intracellular calcium in vascular smooth muscle cells [415]. It is also related to retinal vascular diameter and length, which are important in patients with age-related macular degeneration, as this decreases and narrows during the course of the disease [416,417]. Additionally, it has been reported that some eye diseases, such as glaucoma and macular degeneration, are related to the alteration of calcium-dependent mechanisms, because a high concentration of calcium was an important factor inducing fibrosis, which contributed to fibrotic damage in glaucomatous optic neuropathy [418]. Therefore, it is reasonable to assume that dietary calcium could play an important role in the progression of age-related macular degeneration [418].

## 12. Practical Applications

Dietary habits exert a profound influence on overall health, modulating a plethora of physiological processes. Key mechanisms underlying this relationship encompass oxidative stress, inflammatory cascades, and perturbations in the composition of the gut microbiota, all implicated in the etiology of non-communicable chronic conditions. Within this context, the gut–brain axis emerges as a pivotal mediator, orchestrating bidirectional communication between the enteric nervous system and the central nervous system, thus exerting profound effects on systemic homeostasis. The integration of metabolomics into the framework of nutritional epidemiology presents a promising avenue for elucidating intricate diet–disease interrelations and uncovering novel biomarkers indicative of health status and dietary exposures.

### 12.1. Future Research Directions

In nutrition and the analysis of dietary patterns, nuclear magnetic resonance (NMR) spectroscopy is a robust method which can rapidly analyze mixtures at the molecular level without requiring separation and/or purification steps, making it ideal for applications in food science [419]. Despite its growing popularity among food scientists, NMR remains an underutilized methodology in this field, mainly due to its high cost, relatively low sensitivity, and lack of NMR expertise on the part of many food scientists.

Developing more cost-effective methods for implementing nuclear magnetic resonance (NMR) spectroscopy in the analysis of dietary patterns is essential, thereby enabling the wider adoption of this technique in food science [420].

- Enhancing the sensitivity of NMR spectroscopy to detect molecular components at lower concentrations, expanding its utility in characterizing complex mixtures in foods.
- Designing training and education programs targeting food scientists to increase the understanding and proficiency in NMR spectroscopy, reducing the entry barrier and fostering its use in nutrition and food science research.
- Investigating and developing new analytical techniques and approaches harnessing the advantages of NMR spectroscopy in dietary pattern analysis, such as identifying specific biomarkers of certain diets or assessing food quality and authenticity.
- Promoting interdisciplinary collaboration among food scientists, chemists, physicists, and NMR experts to address technical and scientific challenges associated with the application of NMR spectroscopy in dietary and food research.
- Exploring the integration of NMR spectroscopy with other analytical techniques, such as mass spectrometry, to gain a more comprehensive understanding of food composition and molecular structure and their relationship to human health.
- Establishing collaborations with industry to develop practical applications of NMR spectroscopy in assessing food quality and safety, potentially leading to the creation of innovative tools and technologies for the food industry.
- Investigating the intricate interplay between dietary constituents and the host metabolome to delineate the underlying mechanisms governing disease pathogenesis.
- Examining the longitudinal effects of dietary interventions on metabolic profiles and subsequent health outcomes to inform personalized dietary recommendations.
- Exploring the potential of metabolomics to identify novel biomarkers predictive of dietary adherence and susceptibility to chronic diseases.

### 12.2. Practical Applications

- Encouraging compliance with anti-inflammatory dietary patterns:

Promoting adherence to anti-inflammatory dietary patterns is vital to reduce the risk of chronic diseases like obesity, cardiovascular disease, and diabetes. The Mediterranean diet is a beneficial dietary pattern that focuses on eating fruits, vegetables, whole grains, legumes, nuts, seeds, and olive oil, and reducing intake of red meat and processed foods. Health professionals can decrease inflammation, enhance insulin sensitivity, and minimize the risk of metabolic syndrome by promoting the adoption of the Mediterranean diet. Healthcare practitioners can provide educational programs and culinary workshops centered on Mediterranean food, create personalized meal plans tailored to individual preferences and cultural backgrounds, and offer continuous support and motivation to help individuals sustain dietary changes.

- Executing Specific Dietary Interventions According to Metabolomic Profiling:

Metabolomic profiling is the thorough examination of tiny molecules (metabolites) in biological samples, offering important information about an individual’s metabolic health and risk of disease. Healthcare practitioners can customize dietary therapies to improve metabolic health and slow down disease progression by using metabolomic data. If metabolomic profiling shows lipid metabolism dysregulation, dietary advice may involve consuming more omega-3 fatty acids from fatty fish, flaxseeds, and walnuts, and decreasing intake of saturated and trans fats. If metabolomic data show an impaired glucose metabolism, dietary interventions should prioritize reducing carbohydrate intake by emphasizing complex carbs from whole grains and vegetables rather than simple sweets. Implementing certain dietary changes can improve the management of illnesses such as obesity, type 2 diabetes, and cardiovascular disease.

- Incorporating Metabolomics-Based Biomarkers into Clinical Practice:

Integrating metabolomics-based indicators into clinical practice is crucial for improving the early detection and tailored management of diet-related diseases. Healthcare professionals can improve their assessment of an individual’s risk profile and customize therapies by pinpointing particular metabolites linked to food habits and metabolic disorders. Increased levels of specific amino acids or lipid metabolites in blood samples can suggest a higher likelihood of insulin resistance or cardiovascular disease, leading to more frequent monitoring and dietary adjustments. Healthcare providers can include regular metabolomic profiling into health evaluations, utilizing sophisticated analytical methods to quantify metabolite levels and monitor variations over time. Clinicians can use metabolomics-based biomarkers to identify individuals at risk for diet-related diseases and tailor individualized dietary recommendations for improved health outcomes.

In summary, the convergence of metabolomics and nutritional epidemiology holds immense promise in advancing our understanding of diet–health relationships, offering novel insights into disease prevention and management strategies.

## 13. Conclusions

Diet is a key factor in the prevention and treatment of various chronic diseases that affect people’s health. These diseases include cardiovascular diseases, psychological and psychiatric disorders, oral and visual diseases, metabolic disorders, and cancer. These diseases have in common the involvement of inflammatory and oxidative processes that can be modulated by the nutrients we consume. It is interesting to find easily applicable solutions in dietary intake that allow for an improvement in both the physical and mental quality of life. This type of approach should be multifactorial and consider the biochemical profile of patients, seeking nutrients that modulate the entire body and adapting to individual needs.

## Data Availability

Not applicable.
